# Postoperative Renal Outcomes of Patients Receiving Percutaneous Nephrolithotomy versus Pyelolithotomy: A Population-Based Cohort Study

**DOI:** 10.1155/2018/8582901

**Published:** 2018-05-02

**Authors:** Fang-Ting Chen, Fu-Chao Liu, Chih-Wen Cheng, Jr-Rung Lin, Huang-Ping Yu

**Affiliations:** ^1^Department of Anesthesiology, Chang Gung Memorial Hospital, Taoyuan, Taiwan; ^2^College of Medicine, Chang Gung University, Taoyuan, Taiwan; ^3^Clinical Informatics and Medical Statistics Research Center, Chang Gung University, Taoyuan, Taiwan; ^4^Department of Anesthesiology, Xiamen Chang Gung Hospital, Xiamen, China

## Abstract

The aim of this population-based cohort study was to explore postoperative renal outcomes of patients receiving pyelolithotomy versus percutaneous nephrolithotomy (PCNL). Data were retrieved from the Taiwan National Health Insurance Research Database. During the period from Jan 1, 1998, to Dec 31, 2012, there were 2549 and 21654 patients who underwent pyelolithotomy and PCNL, respectively. The postoperative incidence of new diagnosed end stage renal disease (ESRD) was statistically analyzed and compared between the pyelolithotomy and PCNL groups. The perioperative complications of two groups were also analyzed. In comparison to pyelolithotomy, PCNL achieved lower new diagnosed ESRD (1.38% versus 2.28%, *p* = 0.0004). Patients receiving PCNL had significantly higher rates of preoperative hypertension, diabetes mellitus, pulmonary disease, cerebrovascular disease, and coronary artery disease. The hospital stay was shorter in PCNL groups compared with pyelolithotomy groups (8.31 days versus 12.59 days, *p* = 0.0006). In conclusion, PCNL contributed to lower rates of new diagnosed ESRD and hospital stay when compared to pyelolithotomy.

## 1. Introduction

Several methods have been developed for various types of renal stones, depending on the location and distribution. Percutaneous nephrolithotomy (PCNL) and pyelolithotomy are applied for large and/or complex renal calculi [1–5]. PCNL are reported to have beneficial effects, including low retreatment rates and a low incidence of complications [6]. However, PCNL has potential limitation in undilated renal system [7]. A serial of comparative studies concerning complications or outcomes between PCNL and pyelolithotomy have been declared [8–11]. As for clinical outcomes, many parameters such as the efficacy and the length of hospital stay and preserved renal function postoperatively were also discussed [12–16].

Among these literatures, there is lacking evidence to emphasize the impact on long term renal outcomes after PCNL or pyelolithotomy. The aim of this cohort study was to analyze postoperative renal outcomes of patients receiving PCNL versus pyelolithotomy. The perioperative complication was also analyzed.

## 2. Materials and Methods

### 2.1. Data Source

We implemented a retrospective, population-based cohort study based on Taiwan's National Insurance Research Database (NHIRD). The deidentified and computerized data was derived from The Bureau of National Health Insurance and firstly established in 1992. Since 1998, the database possesses patient basic information and detailed medical data from medical claims, containing clinical diagnostic codes on the basis of the International Classification of Disease, Revision 9, Clinical Modification (ICD-9-CM).

Access to the NHIRD is limited, just for research purpose, under supervision of the Computer-Processed Personal Data Protection Law and other NHRI regulations. This cohort study was evaluated and approved via the NHIRD research committee and the institutional review board of Chang Gung Memorial Hospital.

### 2.2. Patient Selection and Study Design

**Figure 1:**
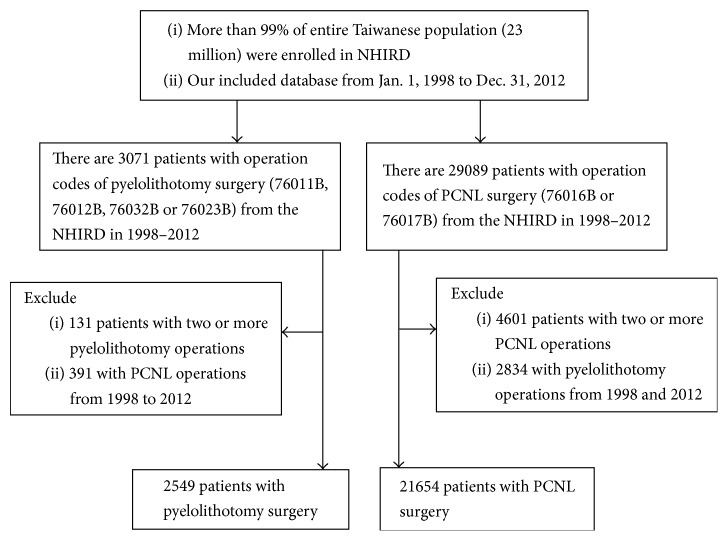
Flow diagram of the study. NHIRD: National Insurance Research Database; PCNL: percutaneous nephrolithotomy.

Figure 1 presents the flowchart of patient identification and selection. Patients receiving PCNL or pyelolithotomy surgery to remove larger/complex urinary stones were identified from the NHIRD with the ICD-9-CM codes. With regard to PCNL, ICD-9-CM operation codes 76016B (PCNL) and 76017B (nephroscope including secondary surgical operation of PCNL) were utilized for identification. Otherwise, ICD-9-CM operation codes 76011B (nephron-pyelolithotomy), 76012B (stag-horn nephron-pyelolithotomy), 76032B (retroperitoneoscopy, laparoscopy, and pyelolithotomy), and 76023B (anatrophic nephrolithotomy) were applied to recognize pyelolithotomy. Over the period from January, 1998, to December, 2012, there were 21654 patients who underwent PCNL and 2549 patients who underwent pyelolithotomy only once, respectively.

Preoperative medical comorbidities were identified from diagnosis in outpatient departments (OPD) or inpatient departments (IPD). All diagnoses were verified with the ICD-9-CM codes as follows: hypertension (ICD-9-CM 401-405), diabetes mellitus (ICD-9-CM 250, A181), pulmonary diseases (ICD-9-CM 490-496, A323, A325), cerebrovascular disease (ICD-9-CM 430-438, A291-299), coronary heart disease (ICD-9-CM 410-414, A279), congestive heart failure (ICD-9-CM 428, A289), vascular disease (ICD-9-CM 443, 444, A302), chronic hepatitis (ICD-9-CM 070, 571, 573.3, A347), and chronic renal failure (ICD-9-CM 585).

### 2.3. Measurement

Our primary outcome was to estimate of incidence of new onset end stage renal disease (ESRD) after receiving PCNL and pyelolithotomy during the period from 1998 to 2012, which was followed till 2013. The primary outcome was compared between patients undergoing PCNL and pyelolithotomy. The secondary outcome was other adverse postoperative effects, including the total length of hospital stay, bacteremia, postoperative bleeding, and pneumonia.

### 2.4. Statistical Analysis

Between-group differences in the distribution of demographic data, coexisting medical diseases, length of hospitalization, and rates of perioperative complication were estimated using *t* test, chi-squared test, or Fisher's exact test, as appropriate for the type and distribution of the data. The log-rank test was used to examine the differences of postoperative complications between patients of receiving PCNL and pyelolithotomy. The between-group probability of postoperative new onset ESRD was assessed via linear trend of incidence (sum the number of new diagnosed ESRD during the follow-up period) year by year. All analyses were performed using SAS software (version 9.3, SAS Institute Inc, Cary, NC), with a 2-sided *p* value < 0.05 considered to be statistically significant.

## 3. Results

### 3.1. Study Population and Baseline Characteristics

The baseline demographic data from 2549 pyelolithotomy patients and 21654 PCNL patients were shown in Table 1. There was a higher ratio for women to undergo pyelolithotomy (45.12% in pyelolithotomy versus 35.71% in PCNL). According to Table 1, patients in PCNL group were more likely to have preoperative hypertension (38.34% versus 34.41%, *p* = 0.0001), diabetes mellitus (16.14% versus 13.77%, *p* = 0.0019), pulmonary disease (14.41% versus 12.08%, *p* = 0.0014), cerebrovascular disease (8.57% versus 7.22%, *p* = 0.0201), coronary artery disease (11.35% versus 8.59%, *p* < 0.0001), and chronic hepatitis (13.21% versus 8.28%). Nevertheless, in terms of preoperative renal status, there was no difference between these two groups in chronic renal failure (1.69% versus 1.93%, *p* = 0.4036).

### 3.2. Postoperative Outcomes

Table 2 shows the clinical variables identified via univariate analysis thought to be associated with urinary tract stone removal surgeries. The length of hospital stay appears to be longer in pyelolithotomy group (*p* = 0.0006). However, the incidence rates of bacteremia (*p* = 0.1594), pneumonia (*p* = 0.8260), and postoperative bleeding (*p* = 0.2275) did not present significant difference among these two groups.

**Figure 2:**
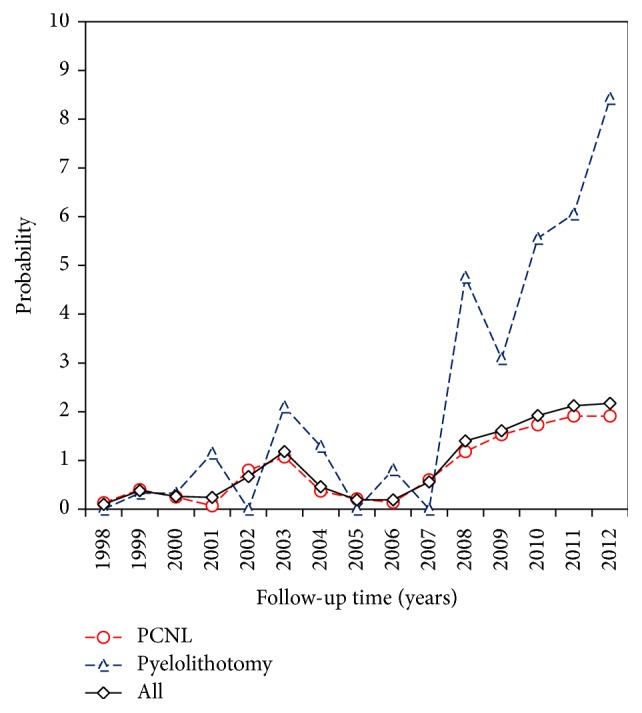
The probability curve of postoperative new diagnosed ESRD following PCNL or pyelolithotomy during follow-up period. ESRD: end stage renal disease; PCNL: percutaneous nephrolithotomy.

New diagnosed ESRD incidence after receiving PCNL or pyelolithotomy was presented in Table 3. It showed that patients who underwent pyelolithotomy had higher risk of postoperative new diagnosed ESRD (2.28% versus 1.38%, *p* = 0.0004). In addition, year probability curve of new diagnosed ESRD was displayed in Figure 2.

## 4. Discussion

This is a retrospective, population-based cohort study to trace the incidence of postoperative ESRD for patients who received a pyelolithotomy or PCNL between 1998 and 2012 and to analyze the associated outcomes. We found that the incidence of new diagnosed postoperative ESRD was higher among patients undergoing pyelolithotomy than PCNL. It also showed that patients receiving pyelolithotomy had a longer hospital stay.

In this cohort study, new diagnosed ESRD patients were defined as having maintenance of hemodialysis or peritoneal dialysis more than 90 days after the first dialysis [17, 18]. Furthermore, those who received hemodialysis would have a frequency ≥26 sessions within 3 months [19]. Hence, we recognize those patients with the following criteria: (1) ICD-9-CM code 585, chronic renal failure, under hemodialysis with any procedure code 58001C, 58027C, or 58029C ≥ 26 times within 3 months and (2) ICD-9-CM code 585, chronic renal failure, under peritoneal dialysis with procedure code 58012B.

On Table 1, there was no significant difference in age distribution between patients receiving pyelolithotomy and PCNL. However, there were more comorbidities such as hypertension, diabetes mellitus, pulmonary disease, coronary artery disease, and cerebrovascular disease among PCNL patients. PCNL was analyzed to be less risky than pyelolithotomy on previous studies [8–11]. Thus, patients with complicated comorbidities might be recommended to receive PCNL rather than pyelolithotomy. That might be the possibility that patients underwent PCNL had more comorbidities in the present study.

Both PCNL and pyelolithotomy might have potential renal injury. In general, pyelolithotomy is considered as a kind of neuron sparing urinary stone removal therapy, causing less parenchyma damage than other techniques [20]. PCNL was also evidenced to have minimal impact on regional or global renal function without significant postoperative alteration [14–16, 21–23]. However, there is lacking cohort study to compare postoperative ESRD between PCNL and open pyelolithotomy. To the best of our knowledge, our study is the first population-based cohort study to evaluate long term renal failure following PCNL versus open pyelolithotomy postoperatively. Our results indicated higher incidence of postoperative new onset of ESRD in open pyelolithotomy patients compared to PCNL patients despite the fact that they had more medical comorbidities.

PCNL is thought to be less invasive and might have lower complication rate than open pyelolithotomy. Nevertheless, it is found that PCNL is still associated with greater kidney functional damage and higher risk for life threatening hemorrhage [24, 25]. On the basis of a hospital-based analysis of pyelolithotomy, the average duration for hospital stay is 3.9 days [12]. As for PCNL, it has even been developed into a kind of ambulatory surgeries recently [26]. However, our data presented much longer hospital stay for both pyelolithotomy and PCNL patients. It might be due to the difference of populations or health care system. The actual reason remains to be determined.

This retrospective population-based cohort study has some potential limitations. The primary consideration is that NHIRD is a secondary database without physical examination and actual medical laboratory data to evaluate the real renal function, such as serum creatinine level and creatinine clearance for further clarification. All of these data may be closely correlated to the impact of surgery on functional renal damage. Thus, we could only identify patients who had end stage renal disease under dialysis with specific codes. However, previous studies had indicated the accuracy and admissibility of ESRD diagnoses in NHIRD [27, 28]. The bias might have minor influence on the final outcome. Secondly, we could not recognize the severity of adverse complications since the severity of perioperative complications cloud is not classified with codes.

In conclusion, PCNL had less deteriorative impact on long term postoperative renal failure compared to pyelolithotomy. Further prospective study is suggested to evaluate the precise mechanism of postoperative long term renal failure after PCNL or pyelolithotomy.

## Figures and Tables

**Table 1:** General demographics of the study subjects.

	Pyelolithotomy		PCNL		*p* value
	(*N* = 2549)		(*N* = 21654)		
Age	54.81 ± 13.31		54.78 ± 12.98		0.9158
Gender		%		%	<0.0001^*∗*^
Female	1150	45.12	7732	35.71	
Male	1399	54.88	13922	64.29	
Hypertension	877	34.41	8302	38.34	0.0001^*∗*^
Diabetes mellitus	351	13.77	3496	16.14	0.0019^*∗*^
Pulmonary disease	308	12.08	3121	14.41	0.0014^*∗*^
Cerebrovascular disease	184	7.22	1856	8.57	0.0201^*∗*^
Coronary heart disease	219	8.59	2457	11.35	<0.0001^*∗*^
Congestive heart failure	58	2.28	580	2.68	0.2295
Vascular disease	19	0.75	245	1.13	0.0759
Chronic hepatitis	211	8.28	2861	13.21	<0.0001^*∗*^
Chronic renal failure	43	1.69	417	1.93	0.4036

Continuous variables were described as the mean ± standard deviation (SD), and the categorical variable was described as number of event (*n*/%); ^*∗*^*p* value < 0.05; PCNL: percutaneous nephrolithotomy.

**Table 2:** Outcome characteristics of patients receiving pyelolithotomy or PCNL.

	pyelolithotomy	PCNL	*p* value
	(*N* = 2549)	(*N* = 21654)	
	Mean (SD)/*n* (%)	Mean (SD)/*n* (%)	
Hospital stay (days)	12.59 (61.68)	8.31(30.20)	0.0006^*∗*^
Bacteremia	80 (3.14)	576 (2.66)	0.1594
Pneumonia	16 (0.63)	144 (0.67)	0.8260
Postoperative bleeding	22 (0.86)	142 (0.66)	0.2275

Continuous variables were described as the mean ± standard deviation (SD), and the categorical variable as number of event (*n*/%); ^*∗*^*p* value < 0.05; PCNL: percutaneous nephrolithotomy.

**Table 3:** Postoperative new diagnosed ESRD and mortality rates of patients receiving PCNL or pyelolithotomy.

	PCNL	%	Pyelolithotomy	%	*p* value
	(*N* = 21654)		(*N* = 2549)		
ESRD	298	1.38	58	2.28	0.0004^*∗*^

^*∗*^ *p* value < 0.05; PCNL: percutaneous nephrolithotomy; ESRD: end stage renal disease.
